# Relationships between hydrophobicity, reactivity, accumulation and peripheral nerve toxicity of a series of platinum drugs

**DOI:** 10.1054/bjoc.1999.1026

**Published:** 2000-02

**Authors:** D Screnci, M J McKeage, P Galettis, T W Hambley, B D Palmer, B C Baguley

**Affiliations:** Department of Pharmacology and Clinical Pharmacology, The University of Auckland, Private Bag 92019, Auckland, New Zealand; School of Chemistry, University of Sydney, Sydney, NSW 2050, Australia; Auckland Cancer Society Research Centre, School of Medicine, The University of Auckland, Private Bag 92019, Auckland, New Zealand

**Keywords:** neurotoxicity, inductively coupled plasma mass spectrometry, structure activity relationships, platinum, cancer chemotherapy

## Abstract

Previous work has shown platinum drugs to differ in their effects on the peripheral nervous system. To test whether their differential toxicity was due to differences in their partitioning into the peripheral nervous system, we correlated the hydrophobicity, reactivity, tissue accumulation and neurotoxicity of a series of eight platinum analogues. Neurotoxicity was detected by measuring sensory nerve conduction velocity (SNCV) in Wistar rats treated twice per week at the maximum tolerated dose. Tissue platinum concentrations were measured by inductively coupled plasma mass spectrometry. Hydrophobicity (log P) was measured using an octanol-aqueous shake-flask method. The half-life of platinum drug binding to plasma proteins in vitro was determined. The cumulative dose causing altered SNCV ranged from 15 to > 2050 μmol kg^−1^. Ranking of the compounds by their neurotoxic potency in rats (oxaliplatin >*R,R* -(DACH)PtC_4_> ormaplatin >*S,S* -(DACH)PtCl_4_>*S,S* -(DACH)Pt oxalato > cisplatin > carboplatin > JM216) correlated with the frequency of neurotoxicity in patients (*r*> 0.99;*P*< 0.05). Ranking the compounds by their peripheral nerve accumulation was cisplatin > carboplatin > oxaliplatin >*R,R* -(DACH)PtCl_4_≈*S,S* -(DACH)PtCl_4_and did not correlate with neurotoxicity. Log P ranged from – 2.53 to –0.16 but did not correlate with neurotoxicity. Log P correlated inversely with platinum accumulation in dorsal root ganglia (r^2^= 0.99;*P* = 0.04), sural nerve (r^2^= 0.85;*P* = 0.025), sciatic nerve (r^2^= 0.98;*P* = 0.0012), spinal cord (r^2^= 0.97, *P* = 0.018) and brain (r^2^= 0.98, *P* = 0.001). Reactivity correlated with neurotoxicity potency in rats (r^2^= 0.89, *P* = 0.0005) and with the frequency of neurotoxicity in patients (r^2^= 0.99, *P* = 0.0002). The hydrophilicity of platinum drugs correlates with platinum sequestration in the peripheral nervous system but not with neurotoxicity. Differences in the reactivity of platinum complexes accounts for some of the variation in their neurotoxicity. © 2000 Cancer Research Campaign


*British Journal of Cancer* (2000) **82**(4), 966–972
© 2000 Cancer Research Campaign
DOI: 10.1054/ bjoc.1999.1026, available online at http://www.idealibrary.com on IDEAL®

### Relationships between hydrophobicity, reactivity, accumulation and peripheral nerve toxicity of a series of platinum drugs

**D Screnci[1], MJ McKeage[1], P Galettis[1], TW Hambley[2], BD Palmer[3] and BC Baguley[3]**

[1]Department of Pharmacology and Clinical Pharmacology, The University of Auckland, Private Bag 92019, Auckland, New Zealand; [2]School of Chemistry, University of Sydney, Sydney, NSW 2050, Australia; [3]Auckland Cancer Society Research Centre, School of Medicine, The University of Auckland, Private Bag 92019, Auckland, New Zealand

**Summary** Previous work has shown platinum drugs to differ in their effects on the peripheral nervous system. To test whether their differential toxicity was due to differences in their partitioning into the peripheral nervous system, we correlated the hydrophobicity, reactivity, tissue accumulation and neurotoxicity of a series of eight platinum analogues. Neurotoxicity was detected by measuring sensory nerve conduction velocity (SNCV) in Wistar rats treated twice per week at the maximum tolerated dose. Tissue platinum concentrations were measured by inductively coupled plasma mass spectrometry. Hydrophobicity (log P) was measured using an octanol-aqueous shake-flask method. The half-life of platinum drug binding to plasma proteins in vitro was determined. The cumulative dose causing altered SNCV ranged from 15 to > 2050 μmol kg$^{-1}$. Ranking of the compounds by their neurotoxic potency in rats (oxaliplatin > *R,R*-(DACH)Pt$C_4$ > ormaplatin > *S,S*-(DACH)Pt$Cl_4$ > *S,S*-(DACH)Pt oxalato > cisplatin > carboplatin > JM216) correlated with the frequency of neurotoxicity in patients ($r > 0.99$; $P < 0.05$). Ranking the compounds by their peripheral nerve accumulation was cisplatin > carboplatin > oxaliplatin > *R,R*-(DACH)Pt$Cl_4$ ≈ *S,S*-(DACH)Pt$Cl_4$ and did not correlate with neurotoxicity. Log P ranged from – 2.53 to –0.16 but did not correlate with neurotoxicity. Log P correlated inversely with platinum accumulation in dorsal root ganglia ($r^2 = 0.99$; $P = 0.04$), sural nerve ($r^2 = 0.85$; $P = 0.025$), sciatic nerve ($r^2 = 0.98$; $P = 0.0012$), spinal cord ($r^2 = 0.97$, $P = 0.018$) and brain ($r^2 = 0.98$, $P = 0.001$). Reactivity correlated with neurotoxicity potency in rats ($r^2 = 0.89$, $P = 0.0005$) and with the frequency of neurotoxicity in patients ($r^2 = 0.99$, $P = 0.0002$). The hydrophilicity of platinum drugs correlates with platinum sequestration in the peripheral nervous system but not with neurotoxicity. Differences in the reactivity of platinum complexes accounts for some of the variation in their neurotoxicity. © 2000 Cancer Research Campaign

**Keywords**: neurotoxicity; inductively coupled plasma mass spectrometry; structure activity relationships; platinum; cancer chemotherapy

Platinum drugs play an important role in the treatment of ovarian (McGuire and Ozols, 1998) and testicular (Einhorn, 1997) cancer but their clinical application can be limited by cumulative damage to the peripheral nervous system (McKeage, 1995). Some platinum derivatives cause frequent or severe symptoms in patients (Gerritsen van der Hoop et al, 1990; Schilder et al, 1994; Machover et al, 1996; O'Rourke et al, 1998), such as numbness or pins and needles in the hands and feet, incoordination and deafness, while other analogues are associated with infrequent or mild neurotoxicity (Canetta et al, 1985; Judson et al, 1997). We have been interested in the mechanism of platinum-induced neurotoxicity and sought to understand why these effects vary between different analogues.

The mechanism of neurotoxicity induced by platinum drugs has been proposed to involve the accumulation of platinum within the peripheral nervous system (Gregg et al, 1992). Evidence in support of this mechanism includes: (1) that exposure to other heavy metals, e.g. lead and mercury, is associated with similar cumulative neurotoxicity and heavy metal accumulation in nervous tissue (Goyer, 1996); (2) that the severity of neurotoxicity associated with cisplatin in patients has been shown to correlate with the concentration of platinum in peripheral nerves (Gregg et al, 1992); and (3) that differences in platinum concentrations between the dorsal root ganglia (DRG) and anterior horn of the spinal cord correlates with the selective toxicity of platinum drugs to peripheral sensory neurons and the sparing of motor neurons from toxicity (Thompson et al, 1984; Gregg et al, 1992).

We hypothesized that the differential neurotoxicity of platinum derivatives may be due to differences in their partitioning into the lipid-rich environment of the nervous system. To test this hypothesis, we measured the hydrophobicity of a series of eight platinum drugs, and in an extension to our previous studies (McKeage et al, 1994; Screnci et al, 1997), we measured the potency of a series of compounds for altering sensory nerve conduction velocity (SNCV) in Wistar rats treated on a twice per week dosing schedule at the maximum tolerated dose (MTD). We measured the concentrations of platinum in various tissues from the nervous system of rats treated with different platinum derivatives using an inductively coupled plasma mass spectrometry (ICP-MS) technique developed in our laboratory (Screnci et al, 1998) and the reactivity of the compounds to plasma proteins. We correlated the experimental parameters and determined whether changes in SNCV in rats predicted neurotoxicity in patients.

*Received 5 July 1999*
*Revised 23 September 1999*
*Accepted 27 September 1999*

*Correspondence to:* MJ McKeage

### MATERIALS AND METHODS

### Drugs (Table 1, Figure 1)

Cisplatin and carboplatin were purchased from Sigma (St Louis, MO, USA). The *R,R*- and *S,S*-enantiomers of ormaplatin ((DACH)$PtCl_4$) and oxaliplatin ((DACH)Pt(oxalato)) were synthesized as outlined previously (Screnci et al, 1997). JM216 was kindly provided by the Johnson Matthey Technology Centre (Reading, UK). The racemic mixture of ormaplatin was kindly supplied by the National Cancer Institute (Bethesda, MD, USA).

### Hydrophobicity measurements

The hydrophobicity (log P) of the series of compounds was determined using a shake-flask method (Albert, 1979). Aqueous (0.9% (w/v) sodium chloride (NaCl) and organic (n-octanol) phases were saturated for 1 week. Drugs were dissolved at a final concentration of 300 μM in the aqueous phase. An equal volume of saturated n-octanol was added and the solutions mixed for 30 min (45 rpm). Samples were centrifuged (1500 ***g***, 10 min) and the platinum content of the organic and aqueous phases determined by ICP-MS. The log of the ratio of platinum concentrations in the organic and aqueous phases were calculated to determine log P.

The hydrophobicity of the series of compounds was also measured using a high-performance liquid chromatography (HPLC) method adapted from a previously reported technique (Braumann, 1986). The HPLC system consisted of a Hewlett Packard HP 1100 HPLC, a Prodigy C8 5 μm column (Phenomenex, Auckland, New Zealand) and a mobile phase consisting of 100% Milli-Q water at a flow rate of 1 ml $min^{-1}$. Methanol was added to the mobile phase for studies of JM216. The HPLC eluent was introduced directly into a Hewlett Packard 4500 ICP-MS. The ICP-MS conditions for detecting platinum were as outlined previously (Screnci et al, 1998).

Compounds were dissolved in 0.9% NaCl at a concentration of 50 μM and injected onto the column (20 μl). Hydrophobicity was measured by determining the capacity factor for each compound when the actual or projected mobile phase composition was 100% water, using the formula; capacity factor = [void volume – retention volume]/void volume. The void volume was determined by detecting the appearance of $Cl^-$ in the HPLC eluent by ICP-MS at mass 35. The retention volume was determined by detecting the presence of platinum in the HPLC eluent by ICP-MS at mass 195.

### Reactivity measurement

Plasma from control female Wistar rats was incubated at 37°C with the platinum compounds at a final concentration of 1 μM. Aliquots of plasma (200 μl) were taken at designated times, added to an equal volume of ice-cold methanol and placed at –20°C overnight. Samples were spun at 38 416 ***g*** for 15 min, an aliquot of supernatant was diluted (tenfold) with 1% nitric acid before analysis by ICP-MS. The initial half-life of binding to plasma proteins was determined by curve fitting (one-phase exponential decay) using Graph Pad Prism (GraphPad Software, CA, USA).

### Animals and treatment

Age-matched inbred female Wistar rats were used. Animals were allowed to acclimatize for 2 weeks before the start of the experiment. The animals were 10 weeks old and weighed 210–240 g at the start of experiment. Platinum compounds (except JM216) were dissolved in sterile 0.9% (w/v) NaCl (Baxter Healthcare, New Zealand) by vortex mixing and sonication at an injection volume of 10 ml $kg^{-1}$. The platinum compounds (except JM216) were given by intraperitoneal injection on a twice per week dosing schedule at intervals of 3–4 days and the treatment was repeated weekly for a total of 8 weeks. JM216 was made up as a suspension in arachis oil and given by oral gavage (McKeage et al, 1994). Animals were weighed twice a week and checked for signs of toxicity daily. Animals showing signs of distress were immediately and painlessly killed. Animals had continuous access to food and water. The project was approved by The University of Auckland Animal Ethics Committee.

### Neurotoxicity assessment

SNCV was calculated from recordings of evoked H-plantar responses before treatment and once a week during treatment as previously described (McKeage et al, 1994) 72 h after platinum drug administration. Hypnorm (fluanisone 10 mg $ml^{-1}$, fentanyl citrate 0.3 mg $ml^{-1}$, Jansen Pharmaceuticals, Sydney, Australia) diluted 1:1 with sterile water was given by intramuscular injection to provide light anaesthesia. Responses were evoked by stimulating the sciatic nerve at the sciatic notch and the tibial nerve at the ankle of the left hind limb using percutaneous needle electrodes. H- and M-waves were recorded using a pair of superficial silver-silver chloride electrodes applied to the sole and dorsum of the hind paw. H- response-related SNCV was calculated by dividing the distance between the stimulation sites at the sciatic notch and ankle by the difference in H-response latency after stimulation at the ankle and sciatic notch. Differences between the means of the control and treatment groups were assessed using a *t*-test. A *P*-value of less than 0.05 was regarded as significant. Neurotoxicity was considered present when there was a statistically significant difference in mean SNCV between the control and treatment groups.

### Tissue platinum determinations

Animals were treated with cisplatin (3.33 μmol $kg^{-1}$), carboplatin (21.6 μmol $kg^{-1}$), oxaliplatin (2.5 μmol $kg^{-1}$) and the *R,R*- (2.6 μmol $kg^{-1}$) and *S,S*- (2.6 μmol $kg^{-1}$) enantiomers of ormaplatin repeated twice per week for up to 8 weeks at the MTD (McKeage et al, 1994; Screnci et al, 1997). Platinum compounds were prepared and given as outlined above.

Three animals were killed by exsanguination under terminal anaesthesia using sodium pentobarbitone (300 mg $kg^{-1}$; Chemstock Animal Health Ltd, Christchurch, New Zealand) at 7, 14, 21, 28, 35, 42, 49 and 56 days for the collection of tissues for platinum analysis. Animals were sacrificed 3 days after the last platinum dose. Blood was collected from the axillary artery into heparinized tubes (Becton and Dickinson, NJ, USA) and plasma was prepared by centrifugation at 3000 ***g*** for 10 min at 4°C. The plasma samples were stored at –20°C until analysis.

Samples of brain, DRG, spinal cord, sural nerve and sciatic nerve were collected. Tissues were washed with Milli-Q water, blotted dry and placed into pre-weighted 15-ml screw cap tubes. Tissues were prepared for ICP-MS analysis as outlined previously (Screnci et al, 1998). Briefly, tissues were left to stand overnight

© 2000 Cancer Research Campaign 

with 1 ml of 70% nitric acid (Riedel-de-Haen, Seelze, Germany). The following day, tissues were digested for 2 h at 90°C in a sand-filled electric frying pan positioned in a fume hood. The solubilized tissues were made to volume in 10-ml volumetric flasks using Milli-Q water and introduced into the ICP-MS. Platinum analysis was undertaken as outlined previously (Screnci et al, 1998) using a HP 4500 (Hewlett Packard) ICP-MS with a nickel sampling cone, V groove nebulizer and a Scott double-pass spray chamber. Platinum was read at 195 amu with a dwell time of 100 ms and a replicate time of 6000 ms. An index of platinum accumulation was derived by calculating the area under the platinum concentration versus time curve (platinum concentration-time product) from days 0–56 using the trapezoid rule and Graph Pad Prism (GraphPad Software Inc., CA, USA).

Blood and plasma platinum determinations were measured by ICP-MS. At the beginning and the end of each run, a matrix-free platinum standard curve was run ranging from 0 to 1000 ng ml$^{-1}$. Platinum standards were made up by diluting a 1000 μg ml$^{-1}$ platinum standard in 10% hydrochloric acid (Spex Certi Prep, USA). Plasma (100 μl) was diluted with 2.4 ml of lysis buffer (0.1% $NH_4$EDTA (Aldrich, Milwalkee, IL, USA), 0.1% Triton X-100 (Serva, Heidelberg, Germany) in 2.5% ammonium hydroxide (BDH Chemicals, Poole, UK)) and introduced into the ICP-MS for analysis.

### Statistical analysis

The significance of relationships between experimental parameters was assessed using linear regression and rank correlation. A two-sided *P*-value of < 0.05 was regarded as statistically significant. Confidence intervals for the incidence of neurotoxicity in patients were calculated using Graph Pad StatMate (GraphPad Software, CA, USA).

### RESULTS

The peripheral neurotoxicity of cisplatin, carboplatin and racemic ormaplatin was assessed in female Wistar rats treated on a twice per week dosing schedule at the MTD. Data were combined with previous results for JM216, oxaliplatin, its *S,S*-enantiomer and the *R,R*- and *S,S*-enantiomers of ormaplatin obtained using the same experimental protocol (McKeage et al, 1994; Screnci et al, 1997). The cumulative dose required to induce altered SNCV in the Wistar rat model ranged from 15 to > 2050 μmol kg$^{-1}$ (Table 1). The number of weeks of treatment required for the development of altered SNCV ranged from 3 to > 20.5 weeks (Table 1).

To provide a relationship between their effects in the Wistar rat model and in patients, clinical toxicity data for cisplatin, carboplatin, oxaliplatin, ormaplatin and JM216 were obtained from the literature (Table 1) (Canetta et al, 1985; Gerritsen van der Hoop et al, 1990; Schilder et al, 1994; Machover et al, 1996; Judson et al, 1997; O'Rourke et al, 1998). The percentage of patients experiencing neurotoxicity during treatment with these platinum drugs ranged from 5.6 to 97%. Ranking of the compounds according to the incidence of neurotoxicity in patients correlated with their ranking by the dose potency for altering SNCV in rats (rank correlation coefficient < –0.99; $P < 0.05$) and by the time required for the development of neurotoxicity in rats (rank correlation coefficient < –0.99; $P < 0.05$).

To measure tissue concentrations of platinum, rats were treated with cisplatin, carboplatin, oxaliplatin (*R,R*-(DACH)Pt(oxalato)) and the enantiomers of ormaplatin (*R,R*-(DACH)$PtCl_4$ and *S,S*-(DACH)$PtCl_4$) twice per week at the MTD. Brain, spinal cord, DRG, sural nerve, sciatic nerve, whole blood and plasma were analysed weekly for platinum content by ICP-MS. There was a time-dependent increase in tissue associated platinum for all drugs. The data for the *R,R*- and *S,S*-enantiomers of ormaplatin are shown in Figure 2. Platinum concentrations in brain and spinal cord were approximately tenfold lower than in DRG, sural nerve and sciatic nerve. Area under the platinum concentration versus time courses from days 0 to 56 (platinum concentration-time products) were calculated as an index of platinum accumulation (Table 2). Ranking the compounds according to their tissue accumulation was similar for all tissues; cisplatin > carboplatin > oxaliplatin > *R,R*-(DACH)$PtCl_4$ ≈ *S,S*-(DACH)$PtCl_4$. However, ranking the compounds according to their plasma platinum concentration-time products was; *R,R*-(DACH)$PtCl_4$ ≈ *S,S*-(DACH)$PtCl_4$ > cisplatin > carboplatin > oxaliplatin. Whole blood platinum concentrations were similar for all drugs.

To provide a relationship between platinum accumulation and neurotoxicity, concentration-time products in tissues, blood and plasma (Table 2) were related to the neurotoxicity of cisplatin,

**Table 1** Ranking of platinum compounds by their peripheral neurotoxicity in female Wistar rats and patients

| Compound | Female Wistar rat | | Patients | |
|---|---|---|---|---|
| | Neurotoxic potency (μmol kg$^{-1}$)[a] | Time to development of altered SNCV (weeks)[b] | Incidence of neurotoxicity in patients % (95% CI) | Reference |
| Oxaliplatin[c] | 15.0 | 3.0 | 97 (92–99) | Machover et al, 1996 |
| *R,R*-(DACH)$PtCl_4$[d] | 17.6 | 4.0 | – | |
| Ormaplatin[e] | 26.0 | 6.0 | 87 (47–100) | Schilder et al, 1994; O'Rourke et al, 1998 |
| *S,S*-(DACH)$PtCl_4$[f] | 30.8 | 7.0 | – | |
| *S,S*-(DACH)Pt-oxalato[g] | 35.0 | 7.0 | – | |
| Cisplatin | 46.7 | 7.0 | 47 (41–53) | Van Der Hoop et al, 1990 |
| Carboplatin | 302.0 | 8.0 | 5.8 (3.8–8.5) | Canetta et al, 1985 |
| JM216 | > 2050.0 | > 20.5 | 5.6 (0–27) | Judson et al, 1997 |

[a]Cumulative dose causing altered SNCV in female Wistar rats during repeated treatment with a twice per week dosing schedule at the MTD. [b]The number of weeks of treatment on a twice per week dosing schedule at the MTD required for the development of altered SNCV. [c]*R,R*-(DACH)Pt(oxalato). [d]*R,R*-enantiomer of ormaplatin. [e]Racemic mixture of *R,R*-(DACH)$PtCl_4$ and *S,S*-(DACH)$PtCl_4$. [f]*S,S*-enantiomer of ormaplatin. [g] *S,S*-enantiomer of oxaliplatin.

© 2000 Cancer Research Campaign

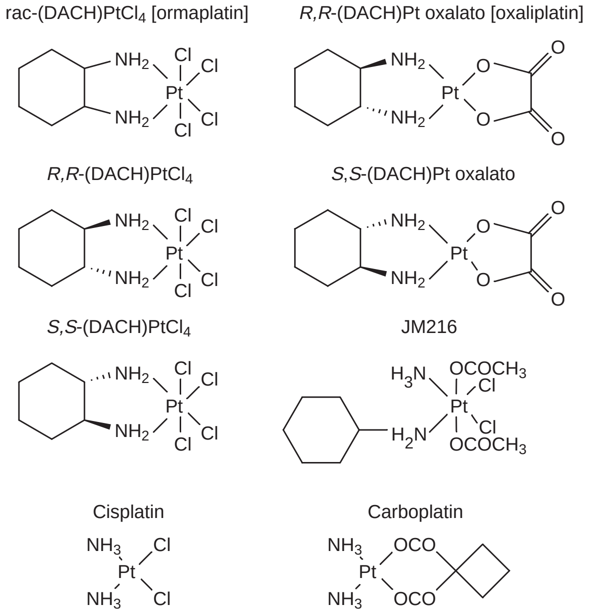

**Figure 1** Chemical structures of platinum complexes carboplatin, oxaliplatin and the *R,R*- and *S,S*-enantiomers of ormaplatin (Table 1). There was no correlation between neurotoxic potency and platinum concentration-time products in DRG ($r^2$ = 0.16; $P$ = 0.57), sural nerve ($r^2$ = 0.01; $P$ = 0.86), sciatic nerve ($r^2$ = 0.18; $P$ = 0.48), brain ($r^2$ = 0.19; $P$ = 0.46), spinal cord ($r^2$ = 0.36; $P$ = 0.28), blood ($r^2$ = 0.14, $P$ = 0.54) or plasma ($r^2$ = 0.23, $P$ = 0.41). At the MTD, cisplatin and carboplatin showed greater tissue concentrations of platinum (Table 2) but less neurotoxicity (Table 1) than oxaliplatin and the isomers of ormaplatin. Animals treated with the *R,R*- and *S,S*-enantiomers of ormaplatin showed similar tissue concentrations of platinum (Figure 2) but a 2.6-fold difference in neurotoxicity (Table 1).

Hydrophobicity of the compounds was measured using an octanol–saline partitioning method (Albert, 1979). Log P values ranged from –2.53 to –0.16 and are shown in Table 3. In attempt to improve the precision of these estimates, another hydrophobicity parameter (log $k_w$) was measured by a reverse-phase HPLC technique adapted from Braumann (1986). Log $k_w$ correlated weakly with log P ($r^2$ = 0.77, $P$ = 0.004). Hydrophobicity ranking of the compounds differed when log $k_w$ was used as a measure of hydrophobicity instead of log P (Table 3). Correlations between hydrophobicity and tissue platinum accumulation were stronger with log P (Figure 3) than log $k_w$.

To determine whether hydrophobicity correlated with neurotoxicity, the relationship between log P and neurotoxicity in rats and patients was assessed by linear regression. Log P did not correlate with neurotoxic potency in rats ($r^2$ = 0.40, $P$ = 0.09), the time to development of neurotoxicity in rats ($r^2$ = 0.34, $P$ = 0.13) or with the incidence of neurotoxicity in patients ($r^2$ = 0.01, $P$ = 0.87). To determine whether the accumulation of platinum was related to hydrophobicity, log P was correlated with platinum concentration-time products in neurological tissues, blood and plasma (Figure 3). Highly significant inverse correlations were found between log P and platinum concentration-time products in DRG ($r^2$ = 0.99; $P$ = 0.004), sural nerve ($r^2$ = 0.85; $P$ = 0.025), sciatic nerve ($r^2$ = 0.98; $P$ = 0.0012), brain ($r^2$ = 0.98, $P$ = 0.0011) and spinal cord ($r^2$ = 0.97; $P$ = 0.0018). Log P did not correlate with platinum concentration-time products in blood ($r^2$ = 0.08; $P$ = 0.64) or plasma ($r^2$ = 0.41; $P$ = 0.25).

The reactivity of cisplatin, oxaliplatin, its *S,S*-enantiomer, ormaplatin, its *R,R*- and *S,S*-enantiomers, and JM216 was determined by measuring their half-lives of binding to plasma proteins in vitro (Table 4). Data were combined with literature values for carboplatin (van der Vijgh and Klein, 1986). The half-life of binding to plasma proteins ranged from 0.78 to 65 h. Reactivity of the platinum compounds correlated with their neurotoxicity potency in rats ($r^2$ = 0.89; $P$ = 0.0005), with the time to development of neurotoxicity in rats ($r^2$ = 0.55; $P$ = 0.036) and with the incidence of neurotoxicity associated with their use in patients ($r^2$ = 0.99; $P$ = 0.0002). There was no correlation between reactivity and platinum concentration-time products or between reactivity and hydrophobicity.

### DISCUSSION

In our previous work, platinum drugs had showed differing effects on the peripheral nervous system in a Wistar rat model (McKeage et al, 1994; Screnci et al, 1997). We now report an attempt to correlate their differential neurotoxicity with their hydrophobicity and tissue accumulation. We found no correlation between the concentration of platinum in the peripheral nervous system and the neurotoxicity of a series of platinum derivatives in a Wistar rat model. We showed that cisplatin and carboplatin were associated with higher concentrations of platinum in peripheral nerves but less neurotoxicity than oxaliplatin and the enantiomers of ormaplatin, at the MTD in rats. Furthermore, the enantiomers of ormaplatin had similar tissue concentrations of platinum but a 2.6-fold difference in neurotoxicity. Another group (Holmes et al, 1998) recently reported their studies on the comparative toxicity of cisplatin, oxaliplatin and ormaplatin to DRG in Wistar rats. They also found cisplatin to be associated with higher concentrations of platinum in

**Table 2** Tissue, blood and plasma platinum concentration-time products (μg $g^{-1}$ $day^{-1}$ or μg $ml^{-1}$ $day^{-1}$) from days 0 to 56 associated with various platinum drugs

| | Cisplatin | Carboplatin | Oxaliplatin | *R,R*-(DACH)$PtCl_4$ | *S,S*-(DACH)$PtCl_4$ |
|---|---|---|---|---|---|
| Dorsal root ganglia | 32.4 | 23.1 | 13.1 | 8.28 | 8.00 |
| Sural nerve | 41.9 | 16.6 | 8.80 | 5.14 | 7.28 |
| Sciatic nerve | 24.9 | 17.8 | 9.65 | 4.38 | 4.83 |
| Brain | 3.98 | 2.93 | 1.43 | 0.94 | 0.97 |
| Spinal cord | 5.52 | 4.96 | 1.72 | 1.08 | 1.11 |
| Blood | 131.0 | 99.9 | 118 | 106 | 97.3 |
| Plasma | 10.6 | 6.17 | 5.45 | 22.7 | 22.4 |

© 2000 Cancer Research Campaign 

**Table 3** Hydrophobicity parameters

| Compounds | Log P[f] | Log $k_w$[g] |
|---|---|---|
| Cisplatin | –2.53 ± 0.28 | –1.00, 0.97 |
| Carboplatin | –2.3 ± 0.10 | 0.20 |
| Oxaliplatin[d] | –1.65 ± 0.21 | 0.44, 0.45 |
| *S,S*-(DACH)Pt oxalato[e] | –1.59 ± 0.21 | 0.44, 0.45 |
| *R,R*-(DACH)$PtCl_4$[b] | –1.18 ± 0.15 | 0.40, 0.42 |
| Ormaplatin[a] | –1.17 ± 0.13 | 0.40, 0.41 |
| *S,S*-(DACH)$PtCl_4$[c] | –1.03 ± 0.14 | 0.41, 0.41 |
| JM216 | –0.16 ± 0.16 | 2.27, 2.40 |

[a]Racemic mixture of *R,R*-(DACH)$PtCl_4$ and *S,S*-(DACH)$PtCl_4$. [b]*R,R*-enantiomer of ormaplatin. [c]*S,S*-enantiomer of ormaplatin. [d]*R,R*-(DACH)Pt oxalato. [e]*S,S*-enantiomer of oxaliplatin. [f]Mean ± standard error ($n$ = 5). [g]Determinants from independent experiments.

**Table 4** Reactivity of platinum complexes measured by the half-life of binding to plasma proteins in vitro

| Compounds | $t_{\frac{1}{2}}$ (h)[a] |
|---|---|
| *S,S*-(DACH)$PtCl_4$[b] | 0.78±0.084 |
| *R,R*-(DACH)$PtCl_4$[c] | 0.97±0.07 |
| Ormaplatin[d] | 1.01±0.15 |
| Oxaliplatin[e] | 1.19±0.10 |
| *S,S*-(DACH)Pt oxalato[f] | 1.21±0.13 |
| Cisplatin | 3.43±0.58 |
| Carboplatin[g] | 48.6 |
| JM216 | 65±27 |

[a]±Standard error. [b]*S,S*-enantiomer of ormaplatin. [c]*R,R*-enantiomer of ormaplatin. [d]Racemic mixture of *R,R*-(DACH)$PtCl_4$ and *S,S*-(DACH)$PtCl_4$. [e]*R,R*-(DACH)Pt oxalato. [f]*S,S*-enantiomer of oxaliplatin. [g]Data from van der Vijgh and Klein, 1986.

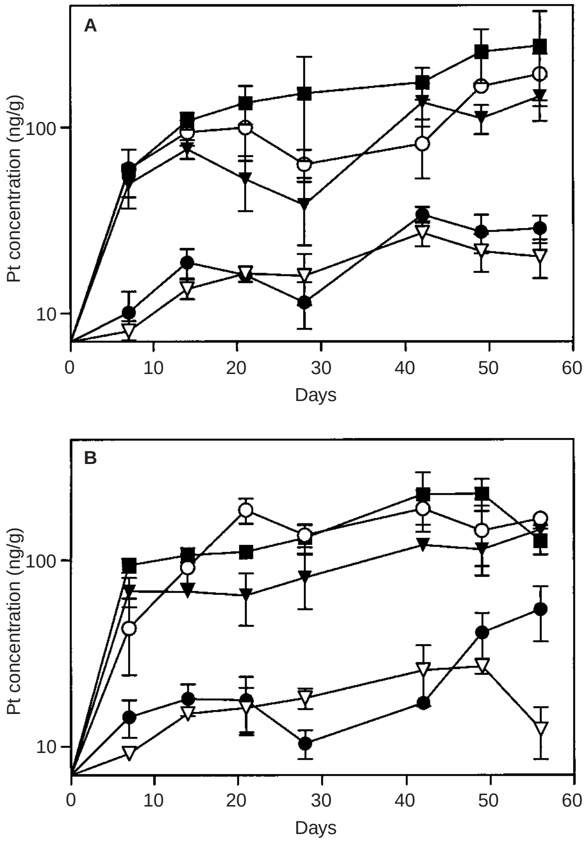

**Figure 2** Time course of concentrations of platinum in DRG (□), sural nerve (○), sciatic nerve (▲), brain (△) and spinal cord (●) during repeated administration of *R,R*- (**A**) or *S,S*- (**B**) enantiomers of ormaplatin twice per week for 8 weeks. Symbols represent the mean and standard error of three replicates

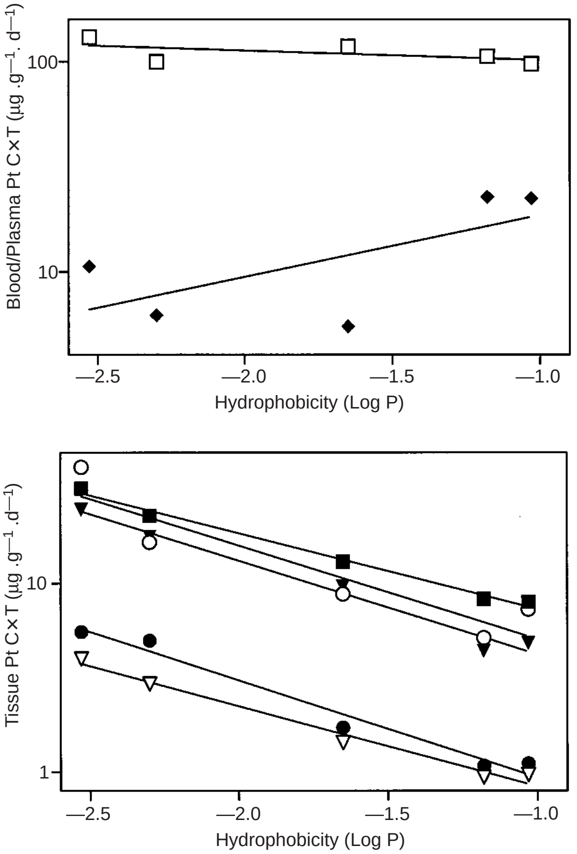

**Figure 3** Relationship between log P of cisplatin (–2.53), carboplatin (–2.30), oxaliplatin (–1.65), *R,R*-(DACH)$PtCl_4$ (–1.18) and *S,S*-(DACH)$PtC1_4$ (–1.03) and platinum concentration-time products (C × T) in whole blood (□), plasma (◆), DRG (■), sural nerve (○), sciatic nerve (▲), brain (△) and spinal cord (●)

DRG than ormaplatin and oxaliplatin, but that the ranking of the compounds according to DRG morphometric change was ormaplatin ≥ oxaliplatin > cisplatin or ormaplatin > cisplatin ≥ oxaliplatin. Taken together, these findings suggest that the peripheral nerve damage caused by platinum drugs involves mechanisms other than the accumulation of elemental platinum within the tissues of peripheral nervous system.

Other workers have found significant relationships between cisplatin dose, the severity of neurotoxicity and the concentration of platinum in the peripheral nervous system in patients treated with cisplatin (Gregg et al, 1992). Patients receiving high cumulative doses of cisplatin experienced greater toxicity and higher tissue concentrations of platinum in this study. Other platinum drugs are likely to show similar relationships between dose, tissue

 *© 2000 Cancer Research Campaign*

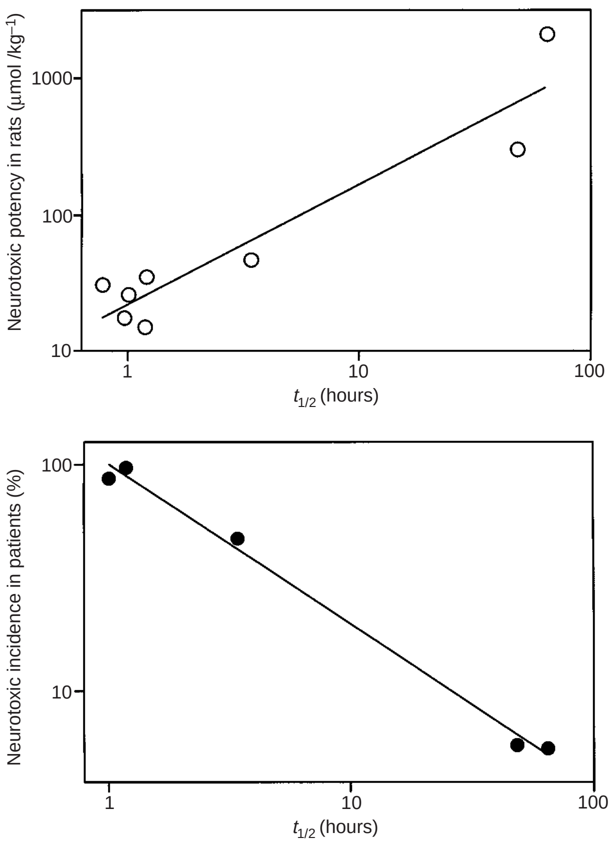

**Figure 4** Relationship between reactivity (half-life of plasma protein binding in vitro) of platinum complexes and neurotoxicity in rats (○) and in patients (●)

concentrations of platinum and neurotoxicity. However, they will differ in the tissue levels of platinum associated with toxicity because their neurotoxicity is not due to the accumulation of elemental platinum in nerves but some intrinsic property of the platinum complex or its biotransformation product(s).

The assay we used to measure tissue platinum (Screnci et al, 1998) did not distinguish between different platinum-containing species or adducts. Platinum drugs are known to form different species in biological systems and to bind to DNA and sulphydryl groups of proteins forming various adducts (Borch, 1987). Their differential neurotoxicity could possibly be due to the formation of different platinum-containing species or adducts within the peripheral nervous system. These differences would not have been detected by the ICP-MS assay because it determined total platinum content and did not differentiate between different platinum-containing species or adducts.

To provide a relationship between hydrophobicity and neurotoxicity, we attempted to measure the octanol–saline partition coefficients (log P) of the compounds using a shake-flask method (Albert, 1979). The results obtained were somewhat variable from day to day. Other workers have found variability in octanol–water partition coefficients determinations possibly due to the formation of micelles within the octanol phase of this partition system (Braumann, 1986; Dearden and Bresnen, 1988). An alternative method was then developed to measure hydrophobicity (log $k_w$) using an HPLC system directly coupled to an element-specific detector (ICP-MS) that detected chloride within the injection solution to determine the void volume and platinum compounds at mass 195. The results were more reproducible from day to day but log $k_w$ correlated only weakly with log P, provided a different hydrophobicity ranking, and predicted the biological distribution of the compounds less well than log P. It may be that the chromotographic conditions used were outside the range where similarities between chromatographic retention, octanol–water partitioning and membrane permeation have been reported to occur (Braumann, 1986). Under these conditions log $k_w$ proved to be an unreliable measure of hydrophobicity of platinum compounds.

The hydrophobicity of the compounds did correlate, however, with the concentration of platinum in DRG, sural nerve, sciatic nerve, spinal cord and brain. The significant inverse correlations between log P and tissue platinum concentration indicated that hydrophilic platinum complexes were associated with higher concentrations of platinum in the peripheral nervous system than lipophilic platinum complexes. The differential tissue accumulation of hydrophilic and lipophilic platinum complexes was not due to differences in their absorption after intraperitoneal injection since there was no correlation between log P and plasma or blood platinum concentration. These findings indicate that hydrophilicity favours the sequestration of platinum in the peripheral nervous system, and suggests that the transport of platinum complexes between the tissue and vascular compartments may involve hydrophilic transport mechanisms such as paracellular transport. It would be interesting to extend this observation to assess whether hydrophilic platinum complexes are preferentially sequested within tumours as well.

The relative reactivity of cisplatin, carboplatin, JM216 and the (DACH)Pt complexes with plasma proteins correlated with their differential peripheral neurotoxicity in the Wistar rat model and in patients. Differences in reactivity, however, did not account for the variation in neurotoxicity shown between *R,R*- and *S,S*-enantiomers of oxaliplatin and ormaplatin. The relative reactivity of platinum compounds with plasma proteins depends upon the rate of formation of aquated reactive electrophilic platinum species (van der Vijgh and Klein, 1986) that then bind to nucleophilic sites, on proteins. It may be that the binding of platinum complexes in, on or outside sensory neurons is involved in the mechanism of peripheral neurotoxicity.

To conclude, in this study the hydrophilicity of platinum drugs correlated with their sequestration within the peripheral nervous system but not with their neurotoxicity. Mechanisms other than the tissue accumulation of platinum must therefore be involved in their neurotoxicity. The relative reactivity of the platinum compounds accounted for some of their differential neurotoxicity. The effects of the series of platinum drugs on SNCV in a Wistar rat model correlated with their neurotoxicity in patients. Neurotoxicity mechanisms can now be investigated in a validated experimental model.

### ACKNOWLEDGEMENTS

This project was supported by the Cancer Society of New Zealand, The Auckland Medical Research Foundation and The Wellcome Trust.

© 2000 Cancer Research Campaign 

### REFERENCES

Albert A (1979) *Selective Toxicity: The Physiochemical Basis Of Therapy*. Chapman and Hall: London

Borch RF (1987) The platinum anti-tumour drugs. In: *Metabolism and Action of Anti-Cancer Drugs*, Powis G and Prough RA (eds), pp. 163–193. Taylor and Francis: London

Braumann T (1986) Determination of hydrophobic parameters by reverse-phase liquid chromatography: theory, experimental techniques and applications in studies on quantitative structure-activity relationships. *J Chromatogr* **373**: 191–225

Canetta R, Rozencweig MO and Carter SK (1985) Carboplatin: the clinical spectrum to date. *Cancer Treat Rev* **12**: (Suppl A) 125–136

Dearden JC and Bresnen GM (1988) The measurement of partition coefficients. *Quant Struct-act Rel* **7**: 133–144

Einhorn IH (1997) Testicular cancer: an oncological success story. *Clin Cancer Res* **3**: 2630–2632

Gerritsen van der Hoop R, van der Burg Mel, Ten Bokkel Huinink WW, van Houwelingen JC and Neijt JP (1990) Incidence of neuropathy in 395 patients with ovarian cancer treated with or without cisplatin. *Cancer* **66**: 1697–1702

Goyer RA (1996) Toxic effects of metals. In: *Casarett and Doull's Toxicology: The Basic Science of Poisons*, Klaassen CD (ed.) pp. 691–736. McGraw-Hill: New York

Gregg RW, Molepa JM, Monpetit VJA, Mikael NZ, Redmond D, Gadia M and Stewart DJ (1992) Cisplatin neurotoxicity: The relationship between dosage, time and platinum concentration in neurological tissues, and morphologic evidence of toxicity. *J Clin Oncol* **10**: 795–803

Judson IR, Cerny T, Epelbaum R, Dunlop D, Smyth J, Schaefer B, Roelvink M, Kaplan S and Hanauske A (1997) Phase II trial of the oral platinum complex JM216 in non-small-cell lung cancer: an EORTC early clinical studies group investigation. *Ann Oncol* **8**: 604–606

McGuire WP and Ozols RF (1998) Chemotherapy for advanced ovarian cancer. *Semin Oncol* **25**: 340–348

McKeage MJ (1995) Comparative adverse effect profiles of platinum drugs. *Drug Saf* **13**: 228–244

McKeage MJ, Boxall F, Jones M and Harrap KR (1994) Lack of neurotoxicity of oral bis-acetato-ammine-dichloro-cyclohexylamine-platinum(IV) (JM216) in comparison to cisplatin and tetraplatin in the rat. *Cancer Res* **54**: 629–631

Machover D, Diaz-Rubio E, de Gramont A, Schilf A, Gastiaburu J, Brienza S, Itzhaki M, Metzger G, N'Daw D and Vignoud J (1996) Two consecutive phase II studies of oxaliplatin (l-OHP) for treatment of patients with advanced colorectal carcinoma who were resistant to previous treatment with fluoropyrimidines. *Ann Oncol* **7**: 95–98

O'Rourke TJ, Weiss GR, New P, Burris HA, Rodriguez G, Eckhart J, Hardy J, Kuhn JG, Fields S and Clark GM (1998) Phase I clinical trial of ormaplatin (tetraplatin NSC 363812). *Anticancer Drugs* **5**: 520–526

Schilder RJ, La Creta FP, Perez RP, Johnson SW, Brennan JM, Rogatko A, Nash S, McAleer C, Hamilton TC and Rody D (1994) Phase I and pharmacokinetic study of ormaplatin (tetraplatin, NSC 363812) administered on a day 1 and 8 schedule. *Cancer Res* **54**: 709–717

Screnci D, Er HM, Hambley TH, Galettis P, Brouwer W and McKeage MJ (1997) Stereo-selective peripheral sensory neurotoxicity of diaminocyclohexane platinum enantiomers related to ormaplatin and oxaliplatin. *Br J Cancer* **76**: 502–510

Screnci D, Galettis P, Baguley BC and McKeage MJ (1998) Optimization of an ICP-MS assay for the detection of trace levels of platinum in peripheral nerves. *Atomic Spectrosc* **19**: 172–175

Thompson SW, Davis LE, Kornfeld M, Hilger RD and Standefer JC (1984) Cisplatin neurotoxicity: clinical, electrophysiologic, morphologic and toxicologic studies. *Cancer* **54**: 1269–1275

van der Vijgh WJF and Klein I (1986) Protein binding of five platinum compounds: comparison of two ultrafiltration systems. *Cancer Chemother Pharmacol* **18**: 129–132

*© 2000 Cancer Research Campaign*
